# Bicarbonate alone does not totally explain the toxicity from major ions of coal bed derived waters to freshwater invertebrates

**DOI:** 10.1007/s10646-022-02552-4

**Published:** 2022-06-14

**Authors:** Kasey A. Hills, Ross V. Hyne, Ben J. Kefford

**Affiliations:** 1 Centre for Applied Water Sciences, Institute for Applied Ecology, University of Canberra, Bruce, ACT 2601 Australia; 2 New South Wales Environmental Protection Authority, Locked Bag 5022, Parramatta, NSW 2124 Australia; 3 Department of Planning, Industry and Environment, Environment Protection Science, Lidcombe Laboratories, Lidcombe, NSW 2141 Australia

**Keywords:** Aquatic invertebrates, Dose–response modelling, Ecotoxicology, Freshwater toxicology, Invertebrate toxicology

## Abstract

Concentrations of major ions in coal mine discharge waters and unconventional hydrocarbon produced waters derived from coal bed methane (CBM) production, are potentially harmful to freshwater ecosystems. Bicarbonate is a major constituent of produced waters from CBM and coal mining. However, little is known about the relative toxicity of differing ionic proportions, especially bicarbonate, found in these CBM waters. As all freshwater invertebrates tested are more acutely sensitive to sodium bicarbonate (NaHCO_3_) than sodium chloride (NaCl) or synthetic sea water, we tested the hypotheses that toxicity of CBM waters are driven by bicarbonate concentration, and waters containing a higher proportion of bicarbonate are more toxic to freshwater invertebrates than those with less bicarbonate. We compared the acute (96 h) lethal toxicity to six freshwater invertebrate species of NaHCO_3_ and two synthetic CBM waters, with ionic proportions representative of water from CBM wells across New South Wales (NSW) and Queensland (Qld), in Australia. The ranking of LC50 values expressed as total salinity was consistent with the hypotheses. However, when toxicity was expressed as bicarbonate concentration, the hypothesis that the toxicity of coal bed waters would be explained by bicarbonate concentration was not well supported, and other ionic components were either ameliorating or exacerbating the NaHCO_3_ toxicity. Our findings showed NaHCO_3_ was more toxic than NaCl and that the NaHCO_3_ proportion of synthetic CBM waters drives toxicity, however other ions are altering the toxicity of bicarbonate.

## Introduction

The concentrations of major ions or salinity is increasing in freshwaters from a range of human activities (Canedo-Arguelles et al. 2013), including the disposal of waste water from- coal bed methane extraction and coal mining (hereafter coal bed waters) (Patz et al. 2004, Veil et al. 2004, Jackson and Reddy 2007, Hamawand et al. 2013, Wright 2012). High salinity levels are toxic to freshwater organisms and can alter ecological interactions (Kefford et al. 2016, Kefford et al. 2012, Bray et al. 2018, Clements and Kotalik 2016). There is concern of adverse effects from disposal of coal bed waters on organisms in streams, their populations, communities, and ecosystems.

The majority of studies that have tested the salinity sensitivity of freshwater organisms have used as a salt source sodium chloride (NaCl)(Jackson and Funk 2019, Blasius and Merritt 2002, Goetsch and Palmer 1997, Palmer et al. 2004, Williams et al. 1999), or Synthetic Marine Salts (SMS) (Kefford et al. 2007, Kefford 2014, Batterton and Baalen, 1971, Dickman and Gochnauer 1978, Gillis 2011, Hills et al. 2019), which is mostly (85%) NaCl. These salts have been widely studied because they are generally representative of salinisation from road de-icing in cold regions (Jackson and Funk 2019, Lob and Silver 2012, Wallace and Biastoch 2016), and dryland salinity in Australia (Sauer et al. 2016).

However, ionic proportions in coal bed waters are dissimilar to those in sea water and do not always have high NaCl levels (Vera et al. 2014). These waters typically have significant concentrations (20–80% of anions) of bicarbonate (HCO_3_^-^) which is much less abundant in sea water (about 0.3% of anions) (Gros et al. 2008). The proportions of ions that make up salinity, may be as important, or even more important, than the total salinity in determining toxicity to freshwater species (O’Neil et al. 1989, Mount and Gulley, 1992, Mount et al. 1997, Cañedo-Argüelles et al. 2016, Mount et al. 2016, Erickson et al. 2018, Mount et al. 2019). Consequently, studies on the toxicity of NaCl and SMS should not be directly used to predict the toxicity of coal bed waters.

There is limited information on the toxicity of NaHCO_3_ but the information available suggests it is more toxic than NaCl and SMS (Hills et al. 2019, Vera et al. 2014, Farag and Harper 2012, Hoke et al. 1992). Hills et al. (2019) determined that eight species of freshwater invertebrates (comprising insects, molluscs, and crustaceans) were two to 50 times more acutely sensitive to NaHCO_3_ than SMS. Moreover, data from the USEPA Ecotoxicology Database (ECOTOX) https://cfpub.epa.gov/ecotox/ showed that six of the nine freshwater animals compared (comprising fish, crustaceans, tubificid worm, mollusc, and insects), were 1.2 to 4.1 times more sensitive to NaHCO_3_ than NaCl (Hills et al. 2019). So, we first hypothesised that coal bed waters containing higher proportions of bicarbonate will be more toxic to freshwater invertebrates than those containing lower proportions of bicarbonate. Conversely, those coal bed waters with higher proportions of chloride compared to bicarbonate, will be relatively less toxic. We secondly hypothesised that the toxicity of coal bed waters would be explained by their bicarbonate concentration.

In this paper, we determine the ionic proportions of coal bed waters collected from 15 mine sites in eastern Australia to categorise similar coal bed waters into groups or ‘Water Types’ with similar ionic proportions. We found two broad water types both with high bicarbonate but one with more chloride relative to the other. We then conducted experiments with these two water types to determine the acute (96 h) mortality of six freshwater invertebrate species. Additionally, we used previously published sodium bicarbonate data (Hills et al. 2019) for the same six species. If our first hypothesis was correct, the water type with relatively more bicarbonate would be more toxic than the water type with less bicarbonate. Further, if our second hypothesis was correct, then there should be no differences in toxicity between the water types when toxicity is expressed as bicarbonate concentration.

## Methods

### Determining which ionic compositions to test

We obtained water chemistry data that the New South Wales (NSW, Australia) Department of Planning, Industry and Environment had collected from 15 mines across five geological basins (Gunnedah, Gloucester, Sydney, Clarence-Moreton, and Murray). This represents five of the six basins in NSW with coal mines, one of these (Clarence-Moreton) crosses into southern Queensland. These data included concentrations of major ions: sodium, potassium, calcium, magnesium, chloride, sulphate, and bicarbonate from 235 samples. Data collected were checked for ionic balance; any sample which was not balanced or did not contain data for all major ions, was removed from the data set.

To determine the effect of coal bed waters on stream invertebrates, we sought to characterise the ionic compositions of these waters, and then determine if they could be grouped into waters with similar ionic compositions or “water types”. Alternatively, the changes in ionic composition from site to site may have rested on a continuous gradient, preventing grouping into water types.

**Fig. 1:**
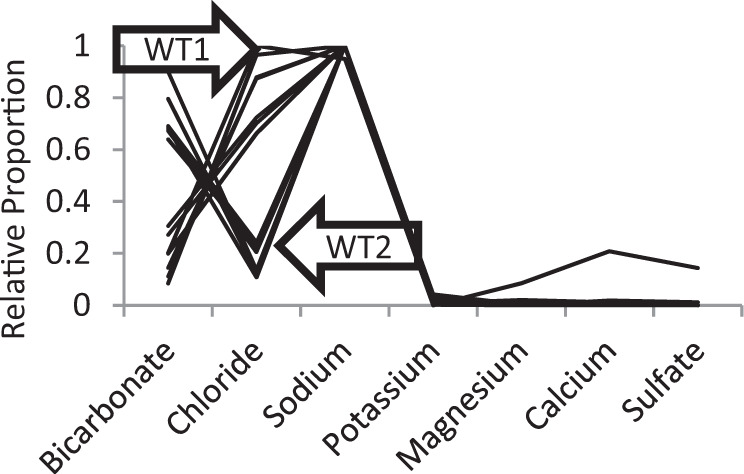
Schoeller Diagram showing the relative proportion of ions within coal bed waters in NSW and Qld. Each line in the graph represents the relative ionic proportions within one of the 15 mining sites. A clear distinction can be seen between waters that are chloride dominant and those that are bicarbonate dominate

To show the relative concentrations of anions and cations within the water, we converted the concentration data (mg/L) into milliequivalents per litre (m equiv/L). As variability between samples from the same site was minimal, the mean concentrations (in m equiv/L) of each ion were then determined for each site. A range-standardisation was then conducted to determine the relative proportions of ions in each of the 15 sites. These range standardisations were then plotted on a Schoeller Diagram (Fig. 1), which are used to plot the relative proportions of ions within the water to help establish whether any patterns exist across sites e.g. (Papendick et al. 2011).

### Preparation of Test Waters

Access to coal bed waters from working mines was not granted, as such water chemistry data that had been previously collected from mine sites, were used to produce synthetic coal bed waters. Two synthetic coal bed waters (WT1 and WT2) were prepared by adding reagent grade (Ajax Finechem, UNIVAR) salts to dechlorinated Sydney tap water. These synthetic waters were made to match the mean proportions identified for Water Types 1 and 2 (Table 1). Prior to use, all solutions were pumped through a 0.45 μm filter (Air-Met Scientific, Sydney, NSW) to remove any particulates or precipitate which may have formed.

**Table 1:** Measured concentrations of ions within 20 g/l stock solutions of both synthetic coal bed waters used within this study

	WT1 (mg/l)	WT2 (mg/l)
Na^+^	6100	6500
Cl^−^	8200	1900
HCO_3_^−^	4100	11200
Ca^2+^	23	10
Mg^2+^	30	25
K^+^	52	140
SO_4_^2−^	8	7
Ca:Mg	0.77	0.40
EC	26.9 mS/cm	18.6 mS/cm

### Animal collection and transport

We prioritised testing of *P. australiensis* because it was the most sensitive to sodium bicarbonate of eight species tested (Hills et al. 2019), *C. dubia* because it is a standard test species and *A. pusillus* because it is a highly salt sensitive mayfly (Kefford 2019, Dowse et al. 2017). Other species (i.e. *C. destructor*, *J. kutera*., and *I. newcombi*) were not able to be tested to all water types but were included to increase the generality of our findings across species.

Three of the six species studied were collected from near Sydney, NSW, Australia: *A. pusillus* and *J. kutera*, were collected from Rouchel Brook (32°8′15.40″S, 150°59′51.16″E) and the Hunter River (31°55′33.12″S, 151°14′12.82″E), respectively. *P. australiensis* was collected from South West Arm Creek (34°6′54.34″S, 151°4′5.75″E) within the Royal National Park. These three field-collected species were obtained using hand nets after disturbing the bankside vegetation or stream substrate.

The invertebrates were transported to the laboratory in the water from their collection site, in a portable cooler containing nylon mesh and conditioned leaves collected at the site, to provide substrate and food. Water was kept cool using ice placed outside the coolers and the water was aerated. On arrival at the laboratory, the invertebrates were placed in the control water: dechlorinated Sydney tap water (Vera et al. 2014), at the test temperature (20 ± 1 °C), light cycle (14 h day: 10 h night) and light intensity (600–1000 lux). The animals were acclimatised in these conditions for 72 h before commencing toxicity testing.

An additional three species were obtained from non-field sources. Freshwater snails, *I. newcombi*, were obtained from uncontaminated plots at the Yanco Agricultural Institute, in Yanco, NSW. Freshwater crayfish, *C. destructor*, was obtained from a farm located in Karuah, NSW. *C. dubia* was obtained from laboratory bred stock at the Environment Protection Science ecotoxicology laboratory in Lidcombe NSW; originally sourced from the Parramatta River, NSW.

### Acute toxicity tests

Acute 96 h toxicity testing was conducted on native freshwater invertebrates.

*Paratya australiaensis* [Decapoda:Atyidae] *Austrophlebioides pusilis* [Ephemeroptera: Leptophlebiidae], *Ceriodaphnia dubia* [Cladocera: Daphniidae] (48 h only)*, Jappa kutera* [Ephemeroptera: Leptophlebiidae], *Cherax destructor* [Decapoda:Parastacidae] and *Isidorella newcombi* [Hygrophila: Planorbidae].

*A pusilis*, and *J kutera* nymphs were 5–11 mm body length excluding cerci*. C. dubia* were neonates (less than 24 h old). *P. australiensis* were 15–30 mm in length. *C. destructor* were 40–80 mm in length. Mature *I. newcombi* were chosen.

With the exception of *C. dubia*, which was conducted according to (USEPA 2002), each test was conducted as follows. The test vessels were 1000 ml glass beakers each containing 900 ml of test water, which was not renewed. Each treatment, including the control, consisted of two vessels per test water concentration. This level of replication of treatments/controls (per test) is common in acute lethal toxicity testing (Slaughter et al. 2008). Ten individuals of one species were randomly allocated to each of the test vessels, except for *C. destructor* where five individuals were added. These containers were then randomly placed on a bench, covered with plastic to minimise evaporation, and aeriated. Invertebrates were provided with conditioned leaves for food, shelter, and substrate. Mortality was defined as the cessation of all visible signs of movement or activity, when gently prodded with a probe. Mortality was recorded daily, and any dead animals were removed. An experiment was considered valid if mortality in the control group (pooled two replicates) did not exceed 10% or one individual at the end of the test. All tests conducted were valid.

### Chemical analyses

Major ions in the test waters were determined by ion chromatography at the beginning of the experiments, in accordance with APHA, 4110-B, using a National Association of Testing Authorities, Australia (NATA) accredited commercial laboratory (Envirolab Services, Chatswood, NSW, Australia). Total alkalinity and bicarbonate alkalinity were determined by a double-indicator titration method at the beginning and end of experiments. Osmotic pressure was measured as osmolality (mOsm/kg) using a Fiske Model 210 micro-osmometer (Fiske Associates, Norwood, MA, USA).

### Statistical analysis

All statistical analysis was conducted using SPSS 20. Concentration response curves were generated using Probit regression, with mortality as the dependent variable and total salinity (mg/L), electrical conductivity (EC, in mS/cm), bicarbonate concentration (mg/L), or osmolality (mOsm/kg) (Supplementary material) the independent variable. The conductivity/concentration lethal to 10% of individuals (LC10), 50% of individuals (LC50), and their 95% confidence intervals (CI) were determined from this regression.

Probit regressions were used to produce LC_*x*_ values and concentration response curves based on total salinity, EC, bicarbonate (HCO_3_^−^) concentration, and osmolality values after 96 h. Osmolality data are presented in the supplementary material as it resulted in identical findings to that of EC. NaHCO_3_ toxicity data obtained using the identical methods and calculated in Hills et al. (2019) are presented for comparison purposes.

## Results

### Determining which ionic compositions to test

A distinction could be made between waters dominated by sodium and chloride and waters dominated by sodium and bicarbonate. Of the 15 mines, seven were designated Water Type1 (hereafter WT1) which had relatively high levels of chloride (60–90% of anions) but still had significant amounts of bicarbonate (5–30% of anions). The remaining six were designated Water Type 2 (WT2) and had relatively lower chloride (10–25% of anions) and relatively higher bicarbonate (70–80% of anions). In both water types, sodium was the dominate cation (90–95% of cations). One of the WT2 mines also exhibited a higher proportion of sulphate, calcium, and magnesium than any of the other samples. Given this ionic proportion was relatively rare (≈6% of sites) in our dataset, we will not consider it further (Fig. 1). No pattern was observed in the geographical distributions of the various water types.

### Chemistry of toxicity test waters

No carbonate alkalinity was detected in any of the controls or treatments either at the beginning or end of each experiment. Bicarbonate alkalinity of each treatment solution did not change over the 96 h test. The measured ions (Table 1) in the filtered stock solutions used to make the treatments were reflective of the water types observed from the mine sites (Fig. 1).

### Acute toxicity of WT1 and WT2

**Fig. 2:**
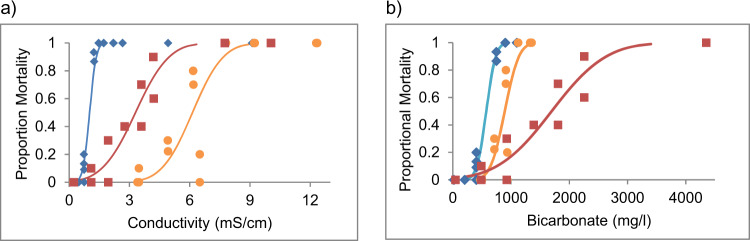
Concentration response curves showing the 96 h toxicity of NaHCO_3_ (blue diamonds), WT1 (yellow circles) and WT2 (red squares) to *P. australiensis* in terms of measured, electrical conductivity (**a**) and measured bicarbonate concentration (**b**)

**Fig. 3:**
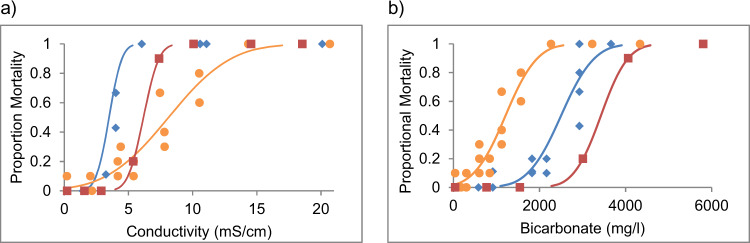
Concentration response curves showing the 96 h toxicity of NaHCO_3_ (blue diamonds), WT1 (yellow circles), and WT2 (red squares) to *A. pusillus* in terms of measured, electrical conductivity (**a**) and measured bicarbonate concentration (**b**)

**Fig. 4:**
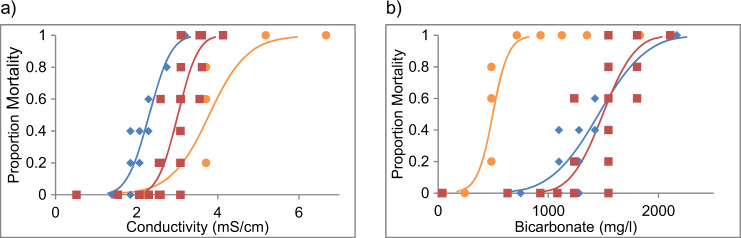
Concentration response curves showing the 48 h toxicity of NaHCO_3_ (blue diamonds), WT1 (yellow circles), and WT2 (red squares) to C. dubia in terms of measured, electrical conductivity (**a**) and measured bicarbonate concentration (**b**)

Regardless of whether salinity is expressed as EC (Table 2), total salinity (i.e. the concentration of all major ions, Supplementary Table S2) or osmolarity (Supplementary Table S1) sodium bicarbonate (Hills et al. 2019) was more toxic than both water types for LC10 and LC50 values and across the concentration response curves (Figs. 2a, 3a and 4a). Again, for EC (Table 2), total salinity (Supplementary Table S2) and osmolarity (Supplementary Table S1), the LC50 values of each water to most species tested followed the prediction that a greater bicarbonate concentration would lead to greater toxicity i.e., WT2 was more toxic than WT1 in all species tested. Except for the mayfly *Jappa sp*., which had a lower LC50 values for WT1 than WT2. For LC10 values, *P. Australiensis* followed the predicted toxicity of WT2 being more toxic than WT1 but there were three species which did not follow this prediction. Both mayfly species (*A. pusillus* and *Jappa sp*) had a lower LC10 for WT1 than WT2 and *C. dubia* had a similar LC10 for both WT1 and WT2.

**Table 2:** Lethal Concentration (LC) values with 96 h exposure to 10% and 50% of the test populations in terms of electrical conductivity (mS/cm @ 25˚C) to three significant figures for taxa tested in this study

	LC10			LC50		
Test Animal	NaHCO_3_	WT1	WT2	NaHCO_3_	WT1	WT2
*Paratya australiensis*	0.67 (0.6–0.8)	4.60 (3.6–5.2)	1.62 (0.8–2.1)	1.01 (0.9–1.1)	6.14 (5.6–6.7)	3.30 (2.9–3.8)
*Austrophlebioides pusillus*	2.48 (1.8–2.9)	3.25 (1.8–4.3)	5.00 (2.6–5.7)	3.49 (3.2–3.9)	8.13 (7.2–9.4)	6.17 (5.3–7.0)
*Ceriodaphnia dubia*	1.75 (1.5–1.9)	2.75 (1.7–3.2)	2.51 (2.3–2.6)	2.31 (2.2–2.4)	3.79 (3.4–4.2)	3.01 (2.9–3.1)
*Cherax destructor*	7.61 (5.6–8.6)	22.3 (16.9–24.8)	NT	10.1 (9.2–11.0)	52.5 (51.5–53.7)	NT
*Isidorella newcombi*	1.28 (0.3–1.9)	4.26 (1.9–5.6)	NT	3.48 (2.9–4.1)	7.69 (6.5–9.0)	NT
*Jappa kutera*	2.84 (2.1–3.2)	6.21 (NR)	8.99 (NR)	4.12 (3.7–4.8)	7.60 (NR)	9.78 (NR)

NaHCO_3_ data from (Hills et al. 2019). Parentheses indicate 95% confidence intervals. Values estimated using probit regression. *NT* Not Tested, *NR* No Result. See Supplementary Table 1 for LC values for 72-h exposure. See also Supplementary Tables S1 and S2 for result in total salinity and osmolarity. See Figs. 2a, 3a and 4a for graphical displays of the concentration response curves for three of these species

Expressing toxicity in terms of bicarbonate concentration (mg/L, instead of salinity) shows a different pattern (Table 3, Figs. 2b, 3b, and 4b). Except for *P. australiensis*, mortality is achieved with a lower concentration of bicarbonate ion in WT1 compared with NaHCO_3_ alone, and a higher concentration of bicarbonate ion in WT2 compared with NaHCO_3_ alone.

**Table 3:** Lethal Concentration (LC) values with 96 h exposure to 10% and 50% of the test populations in terms of bicarbonate (HCO_3_^−^ mg/L) to three significant figures for taxa tested in this study

	LC10			LC50		
Test Animal	NaHCO_3_	WT1	WT2	NaHCO_3_	WT1	WT2
Paratya australiensis	409 (339–460)	647 (455–745)	758 (317–1014)	574 (526–627)	891 (805–979)	1700 (1494–1956)
Austrophlebioides pusillus	1730 (1320–1980)	472 (253–624)	2790 (1499–3171)	2500 (2280–2730)	1210 (1068–1405)	2010 (1658–2444)
Ceriodaphnia dubia	1020 (805–1140)	341 (190–408)	1200 (1095–1275)	1460 (1360–1570)	496 (434–558)	1500 (1436–1556)
Cherax destructor	5860 (4060–6790)	3280 (2398–3725)	NT	8180 (7360–9000)	4450 (4048–4973)	NT
Isidorella newcombi	780 (23.0–1280)	602 (251–803)	NT	2560 (2120–3120)	1130 (945–1332)	NT
Jappa kutera	1820 (913–2220)	963 (NR)	4512 (NR)	2770 (2390–3400)	1190 (NR)	5390 (NR)

NaCHO_3_ data from (Hills et al. 2019). Parentheses indicate 95% confidence interval. Values estimated using probit regression. *NT* Not Tested, *NR* No Result

## Discussion

### Patterns in ionic proportions in coal seam waters

In water collected from 15 mines across five geological basins (Gunnedah, Gloucester, Sydney, Clarence-Moreton, and Murray) ionic proportions were generally consistent with that previously reported in eastern Australia (NSW and Queensland)—that is Surat (Owen et al. 2015, Papendick et al. 2011), Bowen (Kinnon et al. 2010), Clarence-Moreton (Owen et al. 2015), Murray (Shaw 2010) coal basins, as well as the Fitzroy river basin (Mann et al. 2014, Dunlop et al. 2011). The variability in the composition of major ions of groundwater within coal seams at 15 mine sites across coal fields of eastern Australia can be categorised as primarily fitting two water types (Fig. 1).

These water types were:

WT1 (sodium and chloride based)—where sodium and chloride are the dominant cation and anion respectively, and the proportion of bicarbonate (5–30%) being less than chloride (60–90%)

WT2 (sodium and bicarbonate based)—where sodium and bicarbonate are the dominant cation and anion respectively, with the proportions of chloride (10–25%) being less than bicarbonate (70–80%). In both water types, sodium represented 90–95% of the measured cations. All coal bed waters examined for their toxicity had measurable but relatively low calcium (0.03–2.10%), magnesium (0.04–1.39%), potassium (0.20–3.99%), and sulphate (0.00–0.63%) concentrations.

Geochemical processes, including biogenic and thermogenic processes, are typically associated with coal beds and lead to the comparable ionic compositions described above: high sodium, bicarbonate and chloride, and low calcium, magnesium and sulphate (Taulis and Milke 2007). This gives rise to similarity in ionic proportions in the coal beds of other parts of the world, including in the USA (USEPA 2000, Kunz et al. 2013, Veil et al. 2004, Fillo and Evans 1990) and Europe (Van Voast 2003, Hamawand et al. 2013).

### Toxicity of coal seam waters

We evaluated the toxicity of synthetic coal bed waters representing these two Water Types common in eastern Australia, to improve the understanding of their potential impact to the invertebrate community within a stream. Given coal bed waters in the study area vary in ion composition and the specific ionic composition of these waters has the potential to influence the toxicity of these waters to freshwater invertebrates (Mount et al. 1997, Kunz et al. 2013, Cañedo-Argüelles et al. 2016, Mount et al. 2019, Orr et al. 2021). All freshwater species studied were more sensitive to salinity from sodium bicarbonate than NaCl or synthetic marine salts (Hills et al. 2019). We thus hypothesised, that coal bed water toxicity may be driven by bicarbonate proportion and therefore the toxicity of coalbed waters will increase with bicarbonate proportion.

As hypothesised, both water types were less toxic than sodium bicarbonate alone when expressed in terms of salinity (EC, total salinity and osmolarity). Based on LC50 values expressed against salinity; the synthetic coal bed water with the highest proportion of bicarbonate (WT2) was also more toxic than the water with a lower proportion of bicarbonate (WT1), in three of the four species tested. The mayfly *J. kutera*. was the exception, having a lower LC50 values for WT1 than WT2.

However, based on LC10 values, this hypothesis was not well supported with both mayfly species (*A. pusillus* and *J. kutera*) having a lower LC10 for WT1 than WT2, and *C. dubia* having a similar LC10 for both WT1 and WT2. Two of the exceptions, *A. pusillus* and *J. kutera* are mayflies (Ephemeroptera). This insect order is considered to be particularly sensitive to salinity with low bicarbonate concentrations, relative to other insects (Cormier et al. 2008, Cormier et al. 2013, Kefford et al. 2012, Scheibener et al. 2016a, Scheibener et al. 2016b) and the osmoregulatory response of mayflies appears to be somewhat different to other freshwater animals (Kefford 2019, Dowse et al. 2017). *C. dubia* is also known to be sensitive to chloride (Vera et al. 2014).

Furthermore, when toxicity was expressed in bicarbonate concentration (mg/L), the concentration response curves for each water type (NaHCO_3_, WT1 and WT2) do not overlap, which is predicted if bicarbonate is solely driving toxicity in these waters. For *P. australiensis*, which was the most sensitive to bicarbonate, the NaHCO_3_ water was more toxic than WT2, which was more toxic than WT1. This indicates that other ions within the artificial coal bed waters were reducing the toxicity of bicarbonate, or the concentrations of other ions and/or their ratios were otherwise increasing the survivability of *P. australiensis* within these waters (Mount et al. 2016). For other species, WT1 was most toxic in terms of bicarbonate concentration, followed by NaHCO_3_ and then WT2; indicating that other ions (or their ratios) within WT2 may have been having an ameliorating effect, while other ions (or their ratios) in WT1 were increasing toxicity.

The two anions which were the focus of this study chloride, and bicarbonate can both contribute to the toxicity of coal seam waters to freshwater invertebrates (Mount et al. 2016, Kunz et al. 2013, Soucek and Kennedy 2005). Bicarbonate is known to inhibit the uptake of chloride in some freshwater animals (Griffith 2017), potentially leading to a conflict between the toxicity of bicarbonate and the specific concentration of chloride present in the water. Variation of potassium was also observed in the synthetic water types tested (Table 1), with both magnesium and calcium varying in both their concentrations and their ratios. Variations in the magnesium and calcium ratio affects the toxicity of coal bed waters to freshwater invertebrates (Mount et al. 2016, Elphick et al. 2011, Soucek and Dickinson 2015, Soucek and Kennedy 2005). We will explore the potential for chloride, magnesium, calcium, and potassium to alter bicarbonate toxicity in future studies.

In general, when we compare the acute toxicity of each synthetic coal bed water in terms of salinity (as electrical conductivity, total salinity or osmolarity), against each of the three key species tested (Figs. 2, 3, and 4), we can see that relative toxicity generally followed the prediction that a greater bicarbonate concentration would lead to greater toxicity. In each case the LC50 values show that NaHCO_3_ was more toxic than the sodium bicarbonate type water (WT2) which was more toxic than the sodium chloride type water (WT1), although for LC10 values there were exceptions. A coal bed water with higher bicarbonate concentration is acutely toxic to freshwater invertebrates at lower salinity than one with a lower bicarbonate concentration.

The bicarbonate concentration and salinity of both WT1 and WT2 at their respective LC10 and LC50 concentrations determined by these experiments, closely match the bicarbonate concentrations and salinities commonly found in coal bed waters of Australia and the USA. In some cases, bicarbonate concentrations of up to 3.5 g/L have been measured in coal bed waters (Hoke et al. 1992, Papendick et al. 2011, Kinnon et al. 2010). Indicating the potential for these waters to result in direct acute lethality to freshwater invertebrates.

## Supplementary Information

3 Supplimentary Data

## Data Availability

Supplementary material submitted with manuscript. Any further data required can be requested from corresponding author.
