# Supplementary material for: Bicarbonate alone does not totally explain the toxicity from major ions of coal bed derived waters to freshwater invertebrates
Source: Ecotoxicology. 2022 Jun 14;31(6):967–75. doi: 10.1007/s10646-022-02552-4 (PMC9300549; doi:10.1007/s10646-022-02552-4)
Supplement: Supplementary file 1 — 3 Supplimentary Data [file 10646_2022_2552_MOESM1_ESM.docx]

Supplementary Data from: Bicarbonate alone does not totally explain the toxicity from major ions of coal bed derived waters to freshwater invertebrates

| a) | b) |
| --- | --- |

*Figure S1 Dose response curves showing the 96 hour toxicity of NaHCO_3_ (diamonds), WT1 (circles) to Jappa kutera in terms of measured, electrical conductivity (a) and bicarbonate concentration (b)*

| a) | b) |
| --- | --- |

*Figure S2 Dose response curves showing the 96 hour toxicity of NaHCO_3_ (diamonds), WT1 (circles) to Cherax destructor in terms of measured, electrical conductivity (a) and bicarbonate concentration (b)*

| a) | b) |
| --- | --- |

*Figure S3 Dose response curves showing the 96 hour toxicity of NaHCO_3_ (diamonds), WT1 (circles) to Isidorella newcombi in terms of measured, electrical conductivity (a) and bicarbonate concentration (b)*

| a) | b) |
| --- | --- |

Figure S4 Dose response curves showing the 96 hour toxicity of NaHCO3 (diamonds), WT1 (circles) to A. pusillis in terms of, Osmolarity (mOsm/kg) (a) and Salinity (g/L) (b)

| a) | b) |
| --- | --- |

Figure S5 Dose response curves showing the 96 hour toxicity of NaHCO3 (diamonds), WT1 (circles) to C. dubia in terms of, Osmolarity (mOsm/kg) (a) and Salinity (g/L) (b)

| a) | b) |
| --- | --- |

Figure S6 Dose response curves showing the 96 hour toxicity of NaHCO3 (diamonds), WT1 (circles) to C. destructor in terms of, Osmolarity (mOsm/kg) (a) and Salinity (g/L) (b)

| a) | b) |
| --- | --- |

Figure S7 Dose response curves showing the 96 hour toxicity of NaHCO3 (diamonds), WT1 (circles) to I. newcombii in terms of, Osmolarity (mOsm/kg) (a) and Salinity (g/L) (b)

| a) | b) |
| --- | --- |

Figure S8 Dose response curves showing the 96 hour toxicity of NaHCO3 (diamonds), WT1 (circles) to Jappa kutera in terms of, Osmolarity (mOsm/kg) (a) and Salinity (g/L) (b)

| a) | b) |
| --- | --- |

Figure S9 Dose response curves showing the 96 hour toxicity of NaHCO3 (diamonds), WT1 (circles) to P. australiensis in terms of, Osmolarity (mOsm/kg) (a) and Salinity (g/L) (b)

Table S1 Lethal Concentration (LC) values with 96 h exposure to 10% and 50% of the test populations in terms of osmolarity (mOsm/kg) for taxa tested in this study. NaHCO3 data from (Hills et al., 2019). NT = Not Tested, NR = No Result.

|  | LC10 | | | LC50 | | |
| --- | --- | --- | --- | --- | --- | --- |
| Test Animal | NaHCO_3_ | WT1 | WT2 | NaHCO_3_ | WT1 | WT2 |
| *Paratya australiensis* | 12 (10-14) | 81 (58-94) | 35 (18-44) | 18 (16-20) | 114 (103-125) | 68 (60-78) |
| *Austrophlebioides pusillus* | 59 (45-67) | 59 (31-80) | 109 (54-126) | 84 (76-91) | 158 (139-183) | 138 (118-158) |
| *Ceriodaphnia dubia* | 36 (28-40) | 52 (NR) | 48 (44-51) | 50 (47-54) | 60 (NR) | 60 (57-62) |
| *Cherax destructor* | 193 (135-224) | 440 (318-500) | NT | 269 (243-296) | 594 (541-665) | NT |
| *Isidorella newcombi* | 27 (2-43) | 77 (30-103) | NT | 86 (71-104) | 146 (122-173) | NT |
| *Jappa kutera* | 60 (27-74) | 119 (NR) | 182 (NR) | 91 (78-113) | 150 (NR) | 217 (NR) |

Table S2 Lethal Concentration (LC) values with 96 h exposure to 10% and 50% of the test populations in terms of total salinity (g/L) for taxa tested in this study. NaHCO3 data from (Hills et al., 2019). NT = Not Tested, NR = No Result.

|  | LC10 | | | LC50 | | |
| --- | --- | --- | --- | --- | --- | --- |
| Test Animal | NaHCO_3_ | WT1 | WT2 | NaHCO_3_ | WT1 | WT2 |
| *Paratya australiensis* | 0.51 (0.41-0.60) | 2.7 (2.0-3.2) | 1.2 (0.47-1.6) | 0.75 (0.68-0.83) | 3.9 (3.5-4.2) | 2.8 (2.5-3.2) |
| *Austrophlebioides pusillus* | 2.4 (1.8-2.7) | 2.0 (1.0-2.7) | 4.6 (2.2-5.3) | 3.4 (3.1-3.7) | 5.3 (4.7-6.2) | 5.8 (5.0-6.6) |
| *Ceriodaphnia dubia* | 1.4 (1.1-1.6) | 1.7 (NR) | 1.9 (1.8-2.1) | 2.0 (1.9-2.2) | 2.0 (NR) | 2.4 (2.3-2.6) |
| *Cherax destructor* | 8.0 (5.6-9.3) | 15.2 (11.0-17.3) | NT | 11.2 (10.1-12.4) | 20.6 (18.7-23.0) | NT |
| *Isidorella newcombi* | 1.1 (0.0-1.7) | 2.6 (1.0-3.5) | NT | 3.5 (2.9-4.3) | 4.9 (4.1-5.9) | NT |
| *Jappa kutera* | 2.5 (1.1-3.1) | 4.0 (NR) | 7.9 (NR) | 3.8 (3.3-4.7) | 5.1 (NR) | 9.3 (NR) |
